# Precision Medicines for Retinal Lipid Metabolism-Related Pathologies

**DOI:** 10.3390/jpm13040635

**Published:** 2023-04-05

**Authors:** Raquel da Ana, Anna Gliszczyńska, Elena Sanchez-Lopez, Maria L. Garcia, Karolline Krambeck, Andjelka Kovacevic, Eliana B. Souto

**Affiliations:** 1 UCIBIO–Applied Molecular Biosciences Unit, MEDTECH, Laboratory of Pharmaceutical Technology, Department of Drug Sciences, Faculty of Pharmacy, University of Porto, 4050-313 Porto, Portugal; 2 Associate Laboratory i4HB-Institute for Health and Bioeconomy, Faculty of Pharmacy, University of Porto, 4050-313 Porto, Portugal; 3 Department of Food Chemistry and Biocatalysis, Faculty of Biotechnology and Food Science, Wrocław University of Environmental and Life Sciences, Norwida 25, 50-375 Wrocław, Poland; 4 Department of Pharmacy, Pharmaceutical Technology and Physical Chemistry, Faculty of Pharmacy and Food Sciences, University of Barcelona, 08007 Barcelona, Spain; 5 Institute of Nanoscience and Nanotechnology (IN2UB), University of Barcelona, 08007 Barcelona, Spain; 6 Unit of Synthesis and Biomedical Applications of Peptides, IQAC-CSIC, 08034 Barcelona, Spain; 7 Health Sciences School, Guarda Polytechnic Institute, 6300-035 Guarda, Portugal; 8 Department of Pharmaceutical Technology, Institute of Pharmacy, Friedrich Schiller University Jena, 07743 Jena, Germany

**Keywords:** ocular lipid metabolism, lipid oxidation, peroxisomal oxidation, nanoparticles, targeting lipid metabolism

## Abstract

Oxidation of lipids and lipoproteins contributes to inflammation processes that promote the development of eye diseases. This is a consequence of metabolism dysregulation; for instance, that of the dysfunctional peroxisomal lipid metabolism. Dysfunction of lipid peroxidation is a critical factor in oxidative stress that causes ROS-induced cell damage. Targeting the lipid metabolism to treat ocular diseases is an interesting and effective approach that is now being considered. Indeed, among ocular structures, retina is a fundamental tissue that shows high metabolism. Lipids and glucose are fuel substrates for photoreceptor mitochondria; therefore, retina is rich in lipids, especially phospholipids and cholesterol. The imbalance in cholesterol homeostasis and lipid accumulation in the human Bruch’s membrane are processes related to ocular diseases, such as AMD. In fact, preclinical tests are being performed in mice models with AMD, making this area a promising field. Nanotechnology, on the other hand, offers the opportunity to develop site-specific drug delivery systems to ocular tissues for the treatment of eye diseases. Specially, biodegradable nanoparticles constitute an interesting approach to treating metabolic eye-related pathologies. Among several drug delivery systems, lipid nanoparticles show attractive properties, e.g., no toxicological risk, easy scale-up and increased bioavailability of the loaded active compounds. This review analyses the mechanisms involved in ocular dyslipidemia, as well as their ocular manifestations. Moreover, active compounds as well as drug delivery systems which aim to target retinal lipid metabolism-related diseases are thoroughly discussed.

## 1. Introduction

The human retina is an eye structure that is part of the central nervous system, so it shares brain characteristics, such as high metabolism. Indeed, it has the highest mitochondria density of any cell in the body [1,2]. These high energy levels are needed to maintain resting photoreceptors membrane potential, replace photoreceptor outer segments, and combat retinal oxidative stress [1]. It is rich in lipids, which are continuously recycled as a lipid-rich photoreceptor. Lipids and glucose are fuel substrates for photoreceptor mitochondria. In fact, they are important as a membrane component in cells and organelles, but also fuel substrates for the production of energy [2,3]. Lipids are also a fundamental structural component of cell membranes, because of the phospholipid bilayer structure of biological membranes. Cholesterol is a component involved in many different physiological processes and is essential for human development, growth, and physiology. It is composed of a four-ring flanked by a hydroxyl group at carbon 3 of ring A and a branched hydrocarbon side chain at carbon 17 of ring D. The imbalance of cholesterol homeostasis is related to the pathogenesis of AMD, causing irreversible vision loss and blindness. Besides its structural role, mainly in cell membranes, cholesterol is also involved in biochemical processes, as a precursor of steroid hormones, vitamin D, and bile acids, and regulation of gene transcription, and it is also important in nerve conduction and neuronal synapse formation [1,4]. Lipid metabolism is an extremely regulated process, and its dysregulation leads to excess accumulation of lipids in the retinal pigment epithelium (RPE) and Bruch’s membrane (BrM) [5].

Regarding oxidative stress in the human body, it derives from free radicals from reactive oxygen species (ROS), reactive nitrogen species, and reactive carbonyl species, although ROS play the most important role in the oxidative process. Several studies have demonstrated that this is a critical process for increasing or exacerbating many pathological processes. Oxidative stress-related diseases include not only cancer, inflammatory diseases, and diabetes, but also ocular degenerative diseases [6]. Considering the oxygen-rich microenvironment of retina, and that once it receives direct energy from sunlight, it is a photosensitive organ, ROS are easily light-induced. Sunlight energy crosses the cornea, anterior chamber, lens, and vitreous body, and reaches the retina. Ultraviolet (UV) light not only causes DNA damage but, through the production of ROS, also causes photo-oxidative stress. Blue light present in sunlight irradiates lipofuscin granules from RPE and generates ROS, including hydrogen peroxide, superoxide, and singlet oxygen. From the consequences of ROS production, cell damage and aging are described, which result in corneal degeneration, lens opacification (cataracts), and the development of eye diseases, namely various retinal and optic nerve degenerative diseases, such as glaucoma and age-related macular degeneration (AMD) [6,7,8]. In fact, oxidized lipids are among the most important triggers for the development of these pathologies because of the high oxidative stress environment of the macula, the antioxidant capability of RPE that is reduced with aging, and their ability to induce pro-inflammation response [5].

Considering the lipid composition of retina, the outside segment of photoreceptor cells consists of a light-sensitive disc with proteins (mainly photosensitive pigments) and lipids, which include phospholipids and cholesterol, corresponding to 90–95% and 4–6%, respectively, of the total amount of lipids.

Phospholipids are structures with a phosphate head with fatty acid tails that, via cleavage, are dissociated into fatty acids. Considering retinal phospholipids, long-chain polyunsaturated fatty acids (LCPUFA) account for 45% of total phospholipids, saturated fatty acids (SFA) account for around 37%, and monounsaturated fatty acids (MUFA) for approximately 10%. LCPUFA are essential components of ocular structures and functions, and they are part of the processes that cause or resolve inflammation by oxidized lipids [2,9].

In this area, drug delivery systems constitute a useful tool with which to deliver active compounds into the ocular tissues. In recent years, ocular drug delivery systems were investigated to improve ocular retention and drug absorption, as they can work as controlled release systems which aim to reduce administration frequency [10,11,12,13]. Drug delivery systems based on nanocarriers are highly interesting due to their small particle size, low ocular irritation potential and suitable drug availability. Among them, biodegradable nanoparticulated-based systems made of polymers or lipids with mucoadhesive properties are of particular relevance [14,15,16].

In this review, we discuss the mechanisms encountered in ocular dyslipidemia and their biochemical and clinical manifestations. Moreover, active compounds as well as drug delivery systems which aim to target retinal lipid metabolism-related diseases are extensively discussed.

## 2. Dyslipidemia and Its Effects in the Eye

Dyslipidemia is a metabolic dysfunction that refers to an abnormal circulating lipid profile, namely triglycerides, cholesterol, low-density lipoproteins (LDL), high-density lipoproteins (HDL) and polyunsaturated fatty acids [2,17]. It is the main cause of retinal dysfunction and eye diseases, contributing to their set-up and progression. The relation between diabetic retinopathy (DR) and lipid abnormality was found in several studies, showing that high circulation of LDL cholesterol is a significant risk factor for diabetic macular edema and retinal hard exudates [2,3].

The different types of cholesterol form multimolecular complexes with apolipoproteins, phospholipids, and triglycerides (TG) named lipoproteins, to allow their transportation through the systemic circulation and interstitial fluid. Hydrophobic lipids, namely cholesterol ester and TG, are aggregated in the lipoprotein core, which is surrounded by apolipoproteins, which are important to the determination of the lipoproteins pathway, and other polar lipids disposed in a shell, which provide some kind of membrane, allowing their miscibility in plasma. The density and the protein content are inversely related to the particle diameter, giving rise to different classes of particles described above. Therefore, with the decreasing in size of lipoproteins, they also become denser, since there is a loss of lipid content and an increase in proteins [4,17].

Lipid accumulation in the human Bruch’s membrane (BrM), the most inner layer of the choroids, and cholesterol are related to the development of ocular diseases, such as AMD. Lipid-rich lesions associated with this disease (namely drusen, which are small yellow deposits of lipids accumulated in the retina) show significant components such as esterified (EC) and unesterified cholesterol (UC) [4]. Lipid metabolism is regulated by apolipoproteins, structural proteins essential for the metabolism of triglycerides and cholesterol. Lipoproteins found in the posterior ocular segment are part of a distinct class of lipoproteins similar to very low-density lipoproteins (VLDL) concerning size, but they are structurally similar to LDL, as they are rich in esterified cholesterol [5,18]. Apolipoprotein E (ApoE) was identified as a major drusen component. It is a protein component of most lipoproteins, complexes containing lipids and proteins that are responsible for packaging cholesterol and fats from circulation for the transport to tissues and, via reverse cholesterol transport, for removing cholesterol and lipids from tissues. In lipoproteins, ApoE functions to facilitate their cellular uptake, binding to extracellular matrices, thus allowing efficient delivery and removal of lipids from cells. Besides ApoE, other lipid components were found in drusen composition, such as esterified cholesterol, unesterified cholesterol, phosphatidylcholine, triglycerides, sphingomyelin, fatty acids, and other apolipoproteins [8,18]. The oxidation of these lipids and proteins can contribute to a pro-inflammatory environment, promoting the development of AMD. ApoE-containing lipoproteins of drusen come from systemic sources through circulation and from local synthesis by RPE [18]. RPE cells express components for lipoprotein synthesis, ingestion, and secretion, cholesterol synthesis, and reverse cholesterol transport (RCT). In this way, this structure seems to be a central issue in lipid metabolism within the ocular system [5]. Circulating lipoproteins pass through BrM, and this structure also allows the passive diffusion of HDL and LDL [18]. On one hand, systemic circulation of BrM lipoproteins (BrM-LP) seems to be modified by plasma lipoproteins. However, evidence exists that lipoproteins secretion by RPE is a consequence of lipid overload [5]. The increasing plasma levels of HDL and LDL are associated with a higher risk of AMD, since it can be directly related to severe retinopathy and drusen accumulation and growth. Indeed, some studies show that human RPE cells can synthesize and secrete ApoE, which can explain how the locally derived ApoE-containing lipoproteins contribute to drusen biogenesis [18,19,20]. Regarding AMD, HDL are related to their increased risk and drusen development, and triglycerides are associated with a decreased risk of AMD and drusen formation.

Lipid diet modulation also influences diseases with pathological neovascularization, namely retinopathy of prematurity (ROP), AMD, and diabetic retinopathy (DR).

DR is one of the most common diabetes complications and the most common cause of blindness in developed countries [21]. It has been observed that total cholesterol and LDP cholesterol are associated with the presence of hard exudates in patients with DR. Indeed, some studies show the relation between plasma lipids and lipoproteins, namely LDL, and the risk of DR development. [2,19,20]. While the demands of photoreceptor energy lead to vessel growth, photoreceptor-derived oxidative stress and inflammation lead to damage and/or regression of the vascular retina [2].

Moreover, an inverse relationship has been observed between the severity of DR and the amount of ApoA1, whereas ApoB and the ApoB-to-ApoA1 ratio are positively associated with DR. Additionally, lipid-lowering medications seem to be useful as adjunctive therapy for better control of diabetes-related complications, including DR [21].

In the anterior segment, the damage of free radical-induced tissue has been associated with a variety of pathological conditions, such as cataract and experimental autoimmune uveitis [22]. Moreover, lipids can also accumulate in the cornea, causing corneal arcus, which is common in lipid keratopathies [23].

According to the study developed by Módulo et al. (2012) [24], dyslipidemia is also implicated in dry eye disease in preclinical studies. The authors observed that lacrimal gland structure and function are impacted by lipid profile changes in male and female mice. Although not the main cause of dry eye, lipid alterations also seem to contribute to dry eye exacerbation. At the clinical level, it has been reported that there is a significant relationship between total cholesterol and women suffering from dry eye disease. Besides, an association between triglycerides, HDP cholesterol, LDP cholesterol, and dry eye, particularly in women, has also been found [25]. Moreover, alterations due to lipids also seem to be implicated in Meibomian gland disfunction [26]. In this pathology, Meibum cholesteryl esters are decreased, and it has been observed that meibomian gland disfunction patients showed about three times greater prevalence of dyslipidemia (64%) compared to controls (18%) [26,27,28].

## 3. Dysfunctional Peroxisomal Lipid Metabolisms and Their Ocular Manifestations

Peroxisomes are organelles found in all eukaryotic cells and are involved in processes such as α and β-oxidation of lipids, glyoxylate detoxification, amino acid degradation, and metabolism of reactive oxygen species (ROS) and reactive nitrogen species (RNS) [29]. They are also important for the synthesis of plasmalogen, docosahexaenoic acid (DHA), and bile acid [30]. Lipid metabolism and ROS metabolism are both interconnected, and they require the cooperation of peroxisomes with other structures, such as mitochondria, endoplasmic reticulum (RE), lipid droplets, and lysosomes. A great deal of enzymes and transporter proteins are associated with peroxisomes responsible for peroxisome biogenesis, transportation through the peroxisomal membrane, and metabolism [3,31]. Peroxisomes can oxidize fatty acids. Their oxidation requires the activation of fatty acids to a fatty Acyl-CoA [31]. Fatty acid degradation through β-oxidation is a catabolic process that breaks down fatty acid molecules to produce acetyl-CoA, and thus produce energy (ATP) in mitochondria. β-oxidation takes place in the peroxisomes and mitochondria, although ATP is only produced in this last structure. Therefore, peroxisome has a critical role in lipid homeostasis [32,33].

Genetic disorders, with peroxisomal dysfunction, usually lead to different types of ocular complications. Peroxisome degradation breaks down long-chain fatty acids and turns them into shorter-chain fatty acids that, in opposition to the first ones, can be used to produce ATP by mitochondria [2,3].

Considering normal physiology, a balance between peroxisomal biogenesis and degradation is required, together with proper maintenance of peroxisomal function. Otherwise, peroxisomal function is impaired or dysregulated, resulting in a wide variety of age-related diseases [31]. There are two different types of peroxisomal disorders, namely, the disorders of peroxisome biogenesis (PBDs) and the single peroxisomal enzyme deficiencies [34]. Other groups of peroxisomal disorders have so-called multiple enzyme deficiencies. Considering new advances in biochemical and molecular genetic bases, it has also been proposed to split PBD into two subtypes, namely Zellweger Spectrum Disorder (PBD-ZSD) and the rhizomelic chondrodysplasia punctate (RCDP) spectrum. Considering ocular phenotypes related to PBD-ZSD, anterior changes of cornea clouding, cataracts, and Brushfield spots are usually reported. Retinal changes related to this peroxisomal alteration involve all the retinal layers from RPE to retinal ganglion cells, with a great loss of photoreceptors [3]. Besides infantile hypotonia, seizures, and death within the first year of diagnosis, PBD-ZSD causes ophthalmic manifestations, such as corneal opacification, cataract, glaucoma, pigmentary retinopathy, and optic atrophy. Moreover, neonatal adrenoleukodystrophy is associated with a deficiency in a single peroxisomal enzyme and causes cognitive and visual loss that results from demyelination of the entire visual pathway, but the outer retina is spared. Ophthalmic manifestations are especially relevant in peroxisomal-associated disorders, since recognition of the ophthalmic findings is critical for prenatal diagnosis and treatment [35].

Regarding ROS, these can compete for paired electrons of intracellular molecules, which leads to alterations such as lipid peroxidation, protein modification, and chromosomal and mitochondrial DNA lesions. This results in changes in the transmission of information and gene expression, causing autophagy, apoptosis, and necrosis, and triggering tissue and organ dysfunction [6]. Lipid peroxidation is a crucial factor of oxidative stress, causing ROS-induced cell damage and, as mentioned above, it is related to many degenerative diseases such as glaucoma.

LCPUFA are more susceptible to oxidative degradation via lipid peroxidation because of their unsaturated structure, leading to the generation of harmful end-products that strongly contribute to irreversible alterations in cellular components. ROS and oxidized lipoproteins cause cellular stress, which leads to an innate immune response by the activation of cell-associated and soluble pattern recognition receptors (PRRs). In both retinal and choroidal tissues, pro-inflammatory factors (namely TNF-α, IL-1β, and IL-6) are upregulated. An increase in LCPUFA peroxidation products, such as 4-hydroxynonenal (4-HNE), lipofuscin and carboxy ethyl pyrrole (CEP), is also observed, and when they bind to cellular proteins, advanced lipoxidation end products (ALEs) are formed [9]. The accumulation of advanced lipid ALEs affects protein stability, leading to the apoptosis of photoreceptors and RPE cells [19]. Besides, new peroxisomal diseases are related with retinal degeneration, but the pathogenic mechanisms underlying these issues is still unresolved.

## 4. Metabolism Dysregulation in Retinal Diseases and Related Therapies

**Figure 1:**
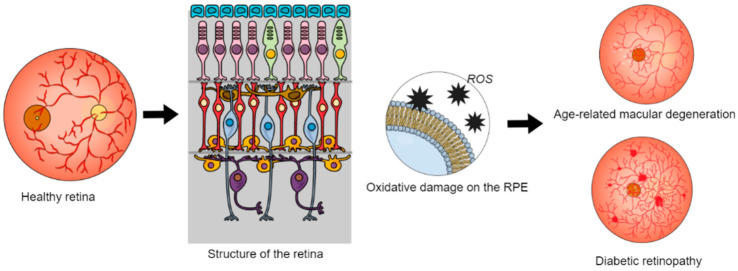
Accumulation of oxidative damage on the RPE causing age-related macular degeneration and contributing to the progression of diabetic retinopathy. (Source: authors’ own drawing).

Retina is one of the human tissues that consume more oxygen. Retinal lipid homeostasis is crucial for the maintenance of normal retinal and vascular function. The accumulation of oxidative damage of RPE is generally caused by the imbalance between the production and elimination of ROS, which is associated with the progression of diabetic retinopathy [36] (Figure 1).

The excessive production of ROS in the retina can be related to the presence of high oxygen metabolism and concentration of polyunsaturated fatty acids and photosensitizers. In this way, the extensive alteration of lipids and peroxisomal dysfunction are great contributors to the development of retinal diseases. The main ROS source is lipofuscin, which, with the aging, is accumulated in the RPE, enhancing oxidative stress in the retina [3,19]. Since lipids are important components for the membrane of cells and organelles, their change in composition leads to the alteration of organelle function and cell status [3].

Moreover, the retinal ganglion cells (RGCs) are located in the most inner layer of the retina—the ganglion cell layer. RGCs constitute the retinal neurons and they extend their axons through the optic nerve to target areas within the brain. This cell group may also be affected by lipid metabolism, causing their apoptosis. For instance, accumulation of GM2 ganglioside in RGCs and other neurons causes a red spot in the fovea. These spots are choriocapillaris surrounded by RGCs with accumulated gangliosides. As a result, RGCs progressively die, causing vision loss [37].

Concerning metabolism-related therapies, three different intervention areas are considered, namely, systemic drug administration, gene therapy, and therapies based on potential targets [1]. Systemic administration of drugs is a challenging issue due to the low efficacy of the delivery for the posterior segment of the eye [38]. The blood–retinal barrier (BRB) is an eye structure that restricts the systemic delivery of some drugs to the retina, mainly hydrophilic and large drugs. Other processes related to drug distribution and clearance result in a short period of action in retina, so higher drug concentrations are needed, which can lead to an increased risk of side effects. In a study on increasing retinal compounds, such as DHA and Neuroprotectin D1 (NPD1), in murine pups with oxygen-induced retinopathy by systemic Arg-Gin, a reduction in pre-retinal neovascularization and restoration of retinal vascular density was reported. This result suggests that systemic administration still has the potential to be used for the treatment of retinal diseases, namely compounds as nutrients, that have a relatively safe profile [1].

## 5. Targeting Lipid Metabolism for the Treatment of Ocular Diseases

The first line of treatment for pathological retinal vessel growth in ocular diseases is anti-vascular endothelial growth factor (VEGF) agents. This approach with anti-VEGF drugs is not totally effective, and has some drawbacks, as the drug remains in systemic circulation for a few months when a single dose is administered by intravitreal injection. Considering the importance of VEGF in neurons and blood vessels, its inhibition can lead to abnormal neurovascular function. In this way, strategies for the prevention and treatment of neovascular retinal diseases should consider the improvement in retinal metabolism, since targeting dysmetabolism-induced inflammatory responses can act as neovascularization suppressors [2]. Indeed, for the treatment of early AMD, lipid metabolism is considered a potential target, and some therapies targeting lipid metabolism are being tested (Table 1), namely desipramine, which prevents ceramide production, DHA, apolipoprotein mimetics, and statins, acting in the reduction of endogenous cholesterol synthesis [1]. Retinal metabolism modulation is a way to restore energy homeostasis to prevent signaling for blood vessel intake, and so prevent neovascularization. It is therefore important to consider interventions that allow us to increase either glucose uptake or fatty acid oxidation and thus to improve energy homeostasis [2].

DHA therapy was also proven to be beneficial and led to visual improvement in DHA-deficient patients with peroxisome biogenesis disorder. This emphasizes the relevant role that a DHA deficiency plays in the retina, especially in peroxisome biogenesis disorder patients, with retinas virtually devoid of DHA. Data indicate that the DHA deficiency is an important pathogenic factor in peroxisomal disorders and should always be corrected. Treatment with DHA ethyl ester, given as early as possible, is strongly recommended, before the damage becomes irreversible [44].

Mouse models have been used to search for new targeting therapies related to lipid metabolism, especially in AMD. Considering AMD preclinical studies, three major therapeutic goals are proposed, namely clearance of pathogenic lipid or protein components in sub-RPE deposits, restoration of lipid processing in RPE and BrM, and finally the preservation of lipid oxidative pathways.

Studies related to the clearance of pathogenic lipid or protein components in sub-RPE deposits, which test therapies to prevent the formation and/or accumulation of Basal laminar deposit (BLamD) in AMD, consider the diet a determinant factor in ocular disease treatment. An in vivo study carried out by Toomey et al. (2015) [45] in mice with the AMD-like phenotype showed that the consumption of a high-fat cholesterol enriched diet increases the circulating lipoproteins. Therefore, complement factor H (CFH) can protect against ocular damage from pathogenic sub-RPE basal deposits once they lack a reservoir of complement components and increase the expression of membrane-bound complement regulators in the posterior eye [45]. One of the therapeutic strategies described for targeting inflammatory lipids in AMD was based on the variation of complement factor H (CFH) activity. CFH is important in the regulation of alternative component pathways, playing a role in the activation and propagation of complement cascade [45]. CFH can also bind and decrease the adherence of ApoE and ApoB-containing lipoproteins to BrM. In this way, it regulates the RPE-derived lipoprotein accumulation in BrM. The results found after the feeding of mice with different types of diet, either rich or low in cholesterol, that showed a correlation of decreased visual function with consumption of dietary cholesterol, support the idea that visual loss in age can be prevented using therapeutic strategies that target cholesterol intake [46]. Considering that it is in the posterior part of the eye that most of the interaction between CFH and HDL occurs, by heparin sulfation within BrM, the authors propose that increasing the CFH concentrations or soluble heparin sulfate in the posterior eye can prevent the accumulation of lipoproteins, namely HDL in BrM, which is a toxic process, and thus protect RPE against damage and death. Pharmaceutical strategies that consider lipoprotein binding in BrM, namely ApoA, which is the major protein constituent of HDL, and associated with AMD risk, seem to be interesting for the treatment of AMD.

One of the most interesting methods used to therapeutically target lipid metabolism is the restoration of pathways that RPE cells use to process lipids. Different transcription factors that participate in intracellular lipid homeostasis are present in RPE cells. Their importance in lipid homeostasis of RPE can be found in the visual loss and lipid deposition in mice because of the lack of liver X receptors (LXR) and peroxisome proliferator-activated receptors (PPAR) signals [18]. PPAR α and γ are both involved in lipid metabolic homeostasis modulation. While PPARα controls triglyceride metabolism and lipoproteins lipase expression, PPARγ upregulates enzymes from fatty acid metabolism, namely the entrance of fatty acid into mitochondria and peroxisome [2]. A study with GW3965, an LXR agonist, was performed to determine if activating LXR has preventive activity in AMD-like development in mice with a mutation in apolipoprotein B100 (ApoB100). The results of the treatment with GW3965 showed an improvement in hypopigmented regions in fundus images, dampening of neuroinflammation, and decreasing lipid deposition [18]. Autophagy is another critical pathway for lipid metabolism. It is induced under stress, as an excess of ROS, to recycle cytosolic components, and thus to remove damaged and dysfunctional cellular components to maintain cellular homeostasis. This is also a fundamental process for the breakdown of photoreceptor outer segments in RPE cells, considering that autophagy-related proteins in retina are present in cellular layers that have a high number of mitochondria because of their high energy needs. In RPE cells of aged non-AMD eyes, autophagy is increased, in contrast with RPE cells from eyes of people with AMD, in which it is decreased. Studies in mice with decreased autophagy showed lipid accumulation within the RPE. As consequence, visual loss, migration of immune cells into subretinal space, and subRPE deposits were observed. In fact, the literature refers to lipofuscin accumulation, reduction in mitochondrial activity, and high levels of ROS as consequences of autophagy deficiency [2,18]. LGM2605 and flubendazole are some of the compounds that are known to induce autophagy, and thus to reduce intracellular RPE lipid levels [18]. Targeting autophagy can therefore be an interesting therapy for AMD treatment.

Another option for intervention in lipid metabolism therapies is to sustain the lipid oxidative function of RPE cells through the preservation of the mitochondria function. As already mentioned, the alterations in mitochondria function have a negative impact on retinal homeostasis. Studies were performed in mice with RPE-specific ablation of mitochondrial transcription factor A (*Tfam*) and specific proteins for mitochondrial biogenesis. This group of mice showed increased activation of the mammalian target of rapamycin (mTOR) pathway, which is essential for nutrient sensing and homeostasis. When the activation of mTOR fails, RPE degeneration can be observed. However, with the inhibition of mTOR in the study group of mice, RPE pathologies are mitigated. In fact, mTOR pathway inhibition through rapamycin can decrease retinal vascularization, leading to hypoxia [18].

## 6. Drug Delivery Systems for Ocular Lipid Metabolism-Related Diseases

**Figure 2:**
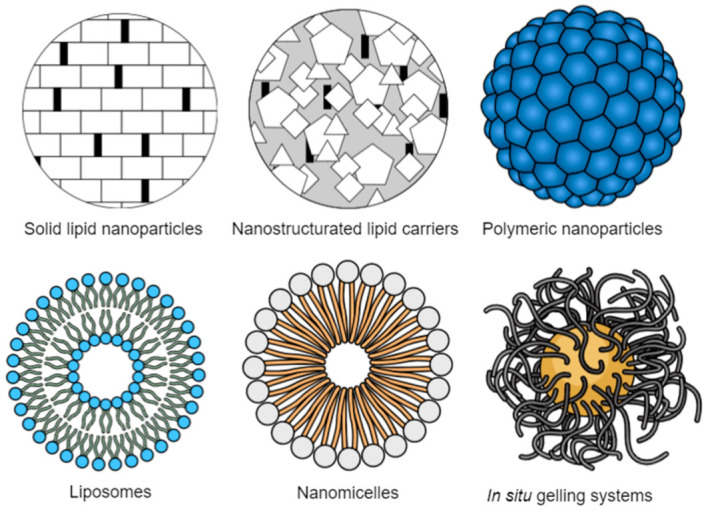
Most recent drug delivery systems used to overcome ocular barriers. (Source: authors’ own drawing).

Ocular drug delivery is one of the most challenging processes in the pharmaceutical field because of the complexity of the eye structure, which impairs drug penetration [38,47]. Ocular drug delivery can essentially be classified into two distinct targets: anterior and posterior segments [38,48]. Although the most conventional administration systems are eye drops, suspensions, and ointments, and correspondingly, around 90% of the ophthalmic formulations found in the market are eye drops, they are not considered effective [47,48]. In fact, topical eye administration is the most popular and well-accepted route, increasing patient compliance. However, ophthalmic drug bioavailability is very low because of the efficient protective mechanisms found in the eye that quickly remove the drug from the eye’s surface. Since only a small amount is available for therapeutic effect, frequent and higher doses are necessary [49]. With this type of formulation used for topic delivery, the drug usually does not reach the posterior segment of the eye, so they are mainly used to target the anterior segment of the eye. Considering the diseases of the posterior segment of the eye, which includes retina, vitreous and choroid tissues, they usually require high drug dosage, with intravitreal administration, implants, or periocular injections [48]. Other administration routes for the treatment of ocular diseases include intracameral, oral, and parenteral administrations [49]. In order to overcome these major challenges and drawbacks related to ocular drug delivery, many new delivery systems have been developed over the last few years (Figure 2).

The main goal of ocular drug delivery is the development of a safe and effective system that delivers the drug on-site, which allows for an increase in the drug bioavailability [47,49]. Some of these new pharmaceutical ophthalmic formulations are in situ gels, different types of nanoparticles, liposomes, nanosuspensions, microemulsions, iontophoresis, and ocular inserts [15,16,50,51]. Nanotechnology-based nanomedicines allow an increase in the residence time, the solubility of lipophilic drugs in an aqueous medium, and the bioavailability and pharmacokinetic properties by enhancing drug penetration in eye barriers [52,53,54,55,56]. Drug encapsulation into nanoparticles also offers the drug physical, chemical, and biological protection against degradation agents [38,47].

In nanosystems for ocular administration, particle size, size distribution and physicochemical stability are some of the critical parameters for the development of a suitable formulation. Therefore, some other advantages of nanoscale drug delivery systems in ocular therapy can be found, namely the possibility of self-administration of eye drops, no impairment of sight due to their small size, the possibility of uptake into corneal cells, and a reduction in side effects and required doses because of the drug targeting effect [38].

### 6.1. Lipid Nanoparticles

Nanosystems, such as microemulsions, self-emulsifying drug delivery systems (SEDDS), liposomes and nanoliposomes, micelles, solid lipid nanoparticles (SLN) and nanostructured lipid carriers (NLC) are all composed of lipids. Lipid nanosystems have interesting properties that allow them to overcome solubility issues related to lipophilic drugs and their poor solubility in tear fluid [50]. Besides this, lipid drug delivery systems are also biodegradable and biocompatible, making them even more interesting in the pharmaceutical field.

SLN and NLC are innovative and promising approaches as drug carriers for ophthalmic applications that can easily be designed for the treatment of the most critical ocular disorders, including those of the retina [10,11]. They are prepared by emulsification of melted lipids, drug, and surfactant in an aqueous medium and subsequent cooling, and show high stability. Additionally, since they are prepared using physiological lipids, the risk of toxicity is very low or non-existent. Moreover, high-pressure homogenization methods are also of special interest to the pharmaceutical industry due to their easy scale-up [57].

SLN and NLC are also particularly interesting because of their wide range of advantages, starting with the increase in bioavailability that results from improving corneal permeation [14,58,59,60]. They are also safe, show no local side effects, and, because of the increased residence time at the administration site, the therapeutic benefits are enhanced without the need for invasive administration [38,50]. SLN and NLC can also be lyophilized and sterilized by heat without relevant effects on stability and in vivo performance [38]. In order to increase the precorneal residence time of lipid nanoparticles and increase their bioavailability, some strategies based on surface modification can be considered [61,62]. The most interesting approaches are the development of a cationic surface that allows the interaction of the particles with the negatively charged mucous layer or epithelium [63]. Coating lipid nanoparticles with mucoadhesive polymers is another strategy to prolong retention time. Some of the most commonly used polymers are polyethylene glycol (PEG) and chitosan, which is a biopolymer [64,65]. Other surface modifications that can be considered are the use of phospholipids, cysteine–polyethylene glycol stearate conjugate, and stearylamine [38,50].

### 6.2. Liposomes

Liposomes are phospholipid-based and bilayered vesicular structures enclosed in an aqueous volume, presenting a highly compatible phospholipid vesicular system. They show the ability to carry both hydrophilic and lipophilic drugs because of their amphiphilic nature. The lipophilic drug is incorporated in the lipid layer, while lipid film formation, and the hydrophilic drug incorporated in the aqueous core, is dissolved in an aqueous medium. Liposomes can be tailored to exhibit targeted delivery properties, a controlled-release profile, and can also be protected from varying pH [66].

Liposomes were developed in 1965, being one of the first lipid systems to be exploited for drug loading and delivery. Additionally, liposomes show several advantages, such as biocompatibility, low toxicological risk, and low antigenicity. They are also biodegradable and metabolized in vivo [67]. Liposomes are one of the most popular nanoparticulate drug delivery systems, which are characterized by nanosized artificial vesicles with single or multiple bilayer membranes and an aqueous core, formed by phospholipids and cholesterol [68,69]. Their limited immunogenicity and high biodegradability profiles are attributed to the phospholipids used, which are similar to lipids found in cell membranes [70,71]. Additionally, they show the capacity to load a wide range of therapeutic compounds of different physicochemical properties, from lipophilic compounds loaded in the lipid bilayers to hydrophilic drugs encapsulated in the aqueous core, and also ionic and amphiphilic molecules that can be loaded using cationic or anionic lipids [72,73]. Liposomes allow us to substantially increase the duration of drug therapeutic action and the drug level in the posterior segment of the eye [74]. They also show high potential for topical eye administration, as well as antibody delivery for the treatment of posterior ocular diseases, due to their high encapsulation efficiency and modified release. The limitations of this system are related to its short shelf life, its frequent need for lyophilization, its limited drug loading capacity, and its lower stability during sterilization, making industrial production more challenging [75,76].

### 6.3. Polymeric Nanoparticles

Polymeric nanoparticles (PNPs) are polymeric colloidal particles, usually classified as nanospheres (if based on a continuous polymeric network in which the drug is uniformly and molecularly dispersed throughout the nanoparticle matrix) or nanocapsules (if based on a core–shell structure in which the drug is dissolved in an oily core surrounded by a polymeric shell) [77]. Lately, PNPs have gained more attention in ophthalmic drug delivery. Different natural and synthetic biocompatible polymers are used for its production, such as poly(lactic-co-glycol acid) (PLGA) and polylactide (PLA). Both undergo degradation in vivo into glycolic acid and lactic acid, respectively, and are easily eliminated [15,78]. Other interesting polymers for nanoparticulate drug delivery systems production are poly(glycolic acid) (PGA), chitosan, Eudragit, gelatin, Carbopol, and others [75,76]. They are particularly interesting because of their biocompatibility, wide range of degradation rates, and absence of toxicity and immunogenicity. Considering the ocular application of PNPs, they not only show good biocompatibility and biodegradability, but also increase drug retention time, providing controlled ocular drug release and sustained therapeutic effects that allow reduced retention time [75,79,80,81].

Besides drug encapsulation, PNPs can also be used for the loading of anti-VEGF agent. Designing PNPs to improve bioavailability of topically applied drugs at the back of the eye is also a targeted search on the development of drug delivery systems, considering the use of oligosaccharides-based NPs [79]. PNPs such as PLA and PLGA nanoparticles can be modified using natural materials, namely chitosan, hyaluronic acid, and cyclodextrin [75,82,83,84]. Chitosan is one of the most used biopolymers, i.e., a hydrophilic polysaccharide with bioadhesive and mucoadhesive properties because of its positive surface charge. Chitosan has been proposed to modify the surface of nanoparticles to increase the residence time on ocular tissues [79]. Besides the relevant properties pointed out for PNPs (e.g., biocompatibility, biodegradability, low toxicity, and antigenicity), some therapeutics containing PLGA have already been approved by the FDA [75]. However, there are some drawbacks, as large particle sizes, namely over 100 nm, depend on the selected materials and production technique. PNPs formulations that consist of a single polymer as a stabilizer also show low physical stability due to uncontrolled particle growth and aggregation. There is thus a real need for the loading of co-stabilizers, even though they show some potential ocular toxicity. Some examples of co-stabilizers (which are widely found in pharmaceutical formulations) include polyvinyl acetate (PVA), polyvinylpyrrolidone (PVP), Pluronics, and cholesterol [76].

Moreover, these nanoparticles can also be functionalized using cell-penetrating peptides, which are able to increase the transport across restrictive barriers and enhance cellular penetration [56,85]. Among several peptides, penetratin and its derivatives have been reported among the most efficient and safe and have been applied for nanoparticle functionalization [86,87,88]. Moreover, antibodies can also be attached to their surface in order to interact with specific ocular moieties [89,90].

### 6.4. Other Biodegradable Nanocarriers

Some other nanoparticulate drug delivery systems developed for eye administration have been successfully described. In fact, it is already possible to find some nanoparticle formulations for targeting retinal diseases in clinical trials, showing the potential of nanoparticles for the management of retinal diseases.

Nanoemulsions are another type of drug delivery system characterized by biphasic dispersion, usually oil-in-water (O/A) emulsions, and low average size (1–100 nm). They are obtained using different surfactants to stabilize the formulation, which can cause eye irritation [74,91,92]. The emulsion droplets are used as a drug reservoir for the encapsulation of both hydrophilic and lipophilic drugs, and their low particle size allows us to increase membrane permeation, enabling drug permeation even to the deeper layers of the eye structure [75,76]. Nanoemulsions are kinetically stable but thermodynamically unstable, and their low viscosity makes them interesting systems for topical administration, allowing us to improve the precorneal residence time [93]. The main limitations of nanoemulsions are related to the risk of toxicity of the surfactants and co-surfactants used for the reduction of interfacial tension, which may cause eye irritation and blurred vision upon eye instillation [75].

Other nanotechnology-based drug delivery systems for ocular application include dendrimers [94,95], nanomicelles [96,97], microparticles [98,99], and inorganic nanoparticles which comprise gold nanoparticles and magnetic nanoparticles [79,100]. Nanomicelles are, indeed, the carriers most frequently used to formulate drugs in clear aqueous media. They are generally made of amphiphilic molecules, namely surfactants or polymers [101]. Some of the properties that make nanomicelles interesting for ocular drug delivery are their high drug encapsulation ability, small size, and easy preparation [102]. Moreover, in situ, gelling systems are another interesting mechanism for ocular drug delivery, and they are related to polymeric solutions that form a viscoelastic gel as a result of environmental responses [16]. This gelation transition can be produced by alterations in temperature, pH, and ions, and by UV radiation [103]. However, considering ocular delivery specifically, research studies are more focused on thermosensitive gels based on temperature changes [100,104,105].

## 7. Conclusions

Considering the consequences of the metabolism dysregulation in the eye, with the development of eye diseases that leads to vision loss in a late stage, such as AMD, it is clear that lipid metabolism in the eye is a crucial issue. Retina is one of the human structures with higher content of lipids, and therefore they are fundamental for its good function. However, an appropriate balance in cholesterol homeostasis is needed, otherwise deposition of lipids in Bruch’s membrane is observed, which, together with other processes such as lipid peroxidation, leads to ROS-induced cell damage. The excessive production of ROS in retina is a recurrent process that depends on the high oxygen metabolism and the concentration of polyunsaturated fatty acids. Changes in lipid metabolism will have effects on lipid oxidation and on lipoproteins, which consequently leads to an increase in the inflammatory process, and ultimately to the development of ocular diseases. It is therefore instrumental to directly target the lipid metabolism when treatingthese diseases, relying on innovative approaches that act in specific sites of the lipid metabolism process, namely, the clearance of pathogenic lipid components found in RPE deposits, restoration of the mitochondria to allow the lipid process in RPE and BrM, and the preservation of lipid oxidative pathways, ensuring a lipid homeostasis balance. Some preclinical tests related to this approach are being performed in mice models with AMD, and the results found show that this is a promising field for the treatment of diseases related to lipid metabolism.

Ocular drug delivery is a challenging topic because of the complexity of the eye structure and the efficacy of its protective mechanisms. Although most of the ocular drug delivery systems present on the market are eye drops, they are not fully efficient. Other systems can be found for the treatment of eye diseases, such as intravitreal injections, periocular injections, implants, and others, depending on each part of the eye that is expected to be targeted. Nanotechnology allows us to overcome the major drawbacks of conventional drug delivery systems, and, considering their unique properties, allow an increase in the safety profile and bioavailability. For most types of nanoparticles, lipid nanoparticles are one of the most interesting approaches to drug delivery, and SLN and NLC have gained more attention. They allow us to increase the residence time on site and decrease the need for high drug doses. They are also prepared using physiologic lipids that are safe and well-accepted by the human body. Additionally, SLN and NLC are more stable than other lipid nanoparticles and enhance physicochemical stability of the encapsulated drug. Lipid nanoparticles seem to be a very interesting approach to the delivery of drugs for the treatment of ocular diseases caused by dysfunctional lipid metabolism.

## Figures and Tables

**Table 1:** Drugs under study for the treatment of metabolic eye diseases (Captions: DR, diabetic retinopathy; AMD, age-related macular degeneration).

Ocular Disease	Compound	Mechanism of Action	References
DR	ALA (Alpha-lipoic acid)	Cofactor for mitochondrial enzyme activity	[39]
DR	Benfotiamine	Coenzyme that acts on the reduction of oxidative stress	[1,40]
DR	FGF21	Regulation of PPARγ	[2]
AMD	GW3965	Activation of LXR	[18,41]
AMD	LGM2605	RPE autophagy inductor with free radical scavenging, antioxidant and anti-inflammatory properties	[18,42]
AMD	Flubendazole	RPE autophagy inductor with increasing lipid degradation accumulation.	[18,43]

## Data Availability

Not applicable.

## References

[1] Chen Y., Coorey N.J., Zhang M., Zeng S., Madigan M.C., Zhang X., Gillies M.C., Zhu L., Zhang T. (2022). Metabolism Dysregulation in Retinal Diseases and Related Therapies. Antioxidants.

[2] Fu Z., Chen C.T., Cagnone G., Heckel E., Sun Y., Cakir B., Tomita Y., Huang S., Li Q., Britton W. (2019). Dyslipidemia in retinal metabolic disorders. EMBO Mol. Med..

[3] Chen C.T., Shao Z., Fu Z. (2022). Dysfunctional peroxisomal lipid metabolisms and their ocular manifestations. Front. Cell Dev. Biol..

[4] Pikuleva I.A., Curcio C.A. (2014). Cholesterol in the retina: The best is yet to come. Prog. Retin. Eye Res..

[5] Jun S., Datta S., Wang L., Pegany R., Cano M., Handa J.T. (2019). The impact of lipids, lipid oxidation, and inflammation on AMD, and the potential role of miRNAs on lipid metabolism in the RPE. Exp. Eye Res..

[6] Hsueh Y.-J., Chen Y.-N., Tsao Y.-T., Cheng C.-M., Wu W.-C., Chen H.-C. (2022). The Pathomechanism, Antioxidant Biomarkers, and Treatment of Oxidative Stress-Related Eye Diseases. Int. J. Mol. Sci..

[7] Choudhary M., Malek G. (2016). Rethinking nuclear receptors as potential therapeutic targets for retinal diseases. SLAS Discov..

[8] Tan L.X., Germer C.J., La Cunza N., Lakkaraju A. (2020). Complement activation, lipid metabolism, and mitochondrial injury: Converging pathways in age-related macular degeneration. Redox Biol..

[9] Ren J., Ren A., Deng X., Huang Z., Jiang Z., Li Z., Gong Y. (2022). Long-Chain Polyunsaturated Fatty Acids and Their Metabolites Regulate Inflammation in Age-Related Macular Degeneration. J. Inflamm. Res..

[10] Sanchez-Lopez E., Espina M., Doktorovova S., Souto E.B., Garcia M.L. (2017). Lipid nanoparticles (SLN, NLC): Overcoming the anatomical and physiological barriers of the eye—Part II—Ocular drug-loaded lipid nanoparticles. Eur. J. Pharm. Biopharm..

[11] Sanchez-Lopez E., Espina M., Doktorovova S., Souto E.B., Garcia M.L. (2017). Lipid nanoparticles (SLN, NLC): Overcoming the anatomical and physiological barriers of the eye—Part I—Barriers and determining factors in ocular delivery. Eur. J. Pharm. Biopharm..

[12] Araujo J., Garcia M.L., Mallandrich M., Souto E.B., Calpena A.C. (2012). Release profile and transscleral permeation of triamcinolone acetonide loaded nanostructured lipid carriers (TA-NLC): In vitro and ex vivo studies. Nanomedicine.

[13] Araujo J., Gonzalez E., Egea M.A., Garcia M.L., Souto E.B. (2009). Nanomedicines for ocular NSAIDs: Safety on drug delivery. Nanomedicine.

[14] Bonilla L., Espina M., Severino P., Cano A., Ettcheto M., Camins A., Garcia M.L., Souto E.B., Sanchez-Lopez E. (2021). Lipid Nanoparticles for the Posterior Eye Segment. Pharmaceutics.

[15] Canadas C., Alvarado H., Calpena A.C., Silva A.M., Souto E.B., Garcia M.L., Abrego G. (2016). In vitro, ex vivo and in vivo characterization of PLGA nanoparticles loading pranoprofen for ocular administration. Int. J. Pharm..

[16] Esteruelas G., Halbaut L., Garcia-Torra V., Espina M., Cano A., Ettcheto M., Camins A., Souto E.B., Garcia M.L., Sanchez-Lopez E. (2022). Development and optimization of Riluzole-loaded biodegradable nanoparticles incorporated in a mucoadhesive in situ gel for the posterior eye segment. Int. J. Pharm..

[17] Barchiesi B.J., Eckel R.H., Ellis P.P. (1991). The cornea and disorders of lipid metabolism. Surv. Ophthalmol..

[18] Landowski M., Rickman C.B. (2022). Targeting Lipid Metabolism for the Treatment of Age-Related Macular Degeneration: Insights from Preclinical Mouse Models. J. Ocul. Pharmacol. Ther..

[19] Deng Y., Qiao L., Du M., Qu C., Wan L., Li J., Huang L. (2022). Age-related macular degeneration: Epidemiology, genetics, pathophysiology, diagnosis, and targeted therapy. Genes Dis..

[20] Busik J.V. (2021). Lipid metabolism dysregulation in diabetic retinopathy. J. Lipid Res..

[21] Chang Y.C., Wu W.C. (2013). Dyslipidemia and diabetic retinopathy. Rev. Diabet. Stud. RDS.

[22] Njie-Mbye Y.F., Chitnis M., Opere C., Ohia S. (2013). Lipid peroxidation: Pathophysiological and pharmacological implications in the eye. Front. Physiol..

[23] Crispin S. (2002). Ocular lipid deposition and hyperlipoproteinaemia. Prog. Retin. Eye Res..

[24] Módulo C.M., Filho E.B.M., Malki L.T., Dias A.C., de Souza J.C., Oliveira H.C.F., Jorge Í.C., Gomes I.B.S., Meyrelles S.S., Rocha E.M. (2012). The Role of Dyslipidemia on Ocular Surface, Lacrimal and Meibomian Gland Structure and Function. Curr. Eye Res..

[25] Rathnakumar K., Ramachandran K., Baba D., Ramesh V., Anebaracy V., Vidhya R., Vinothkumar R., Poovitha R., Geetha R. (2018). Prevalence of dry eye disease and its association with dyslipidemia. J. Basic Clin. Physiol. Pharmacol..

[26] Osae E.A., Steven P., Redfern R., Hanlon S., Smith C.W., Rumbaut R.E., Burns A.R. (2019). Dyslipidemia and Meibomian Gland Dysfunction: Utility of Lipidomics and Experimental Prospects with a Diet-Induced Obesity Mouse Model. Int. J. Mol. Sci..

[27] Borchman D., Ramasubramanian A., Foulks G.N. (2019). Human Meibum Cholesteryl and Wax Ester Variability with Age, Sex, and Meibomian Gland Dysfunction. Investig. Ophthalmol. Vis. Sci..

[28] Knop E., Knop N., Millar T., Obata H., Sullivan D.A. (2011). The international workshop on meibomian gland dysfunction: Report of the subcommittee on anatomy, physiology, and pathophysiology of the meibomian gland. Investig. Ophthalmol. Vis. Sci..

[29] Herman I., Calame D.G. (2021). Clinical and Neuroimaging Features of Peroxisomal Disorders. Neuropediatrics.

[30] Abe Y., Wanders R.J.A., Waterham H.R., Mandel H., Falik-Zaccai T.C., Ishihara N., Fujiki Y. (2022). Genetic defects in peroxisome morphogenesis (Pex11β, dynamin-like protein 1, and nucleoside diphosphate kinase 3) affect docosahexaenoic acid-phospholipid metabolism. J. Inherit. Metab. Dis..

[31] Cipolla C.M., Lodhi I.J. (2017). Peroxisomal Dysfunction in Age-Related Diseases. Trends Endocrinol. Metab..

[32] Lodhi I., Semenkovich C. (2014). Peroxisomes: A Nexus for Lipid Metabolism and Cellular Signaling. Cell Metab..

[33] Binns D., Januszewski T., Chen Y., Hill J., Markin V.S., Zhao Y., Gilpin C., Chapman K.D., Anderson R.G.W., Goodman J.M. (2006). An intimate collaboration between peroxisomes and lipid bodies. J. Cell Biol..

[34] Wanders R. (2004). Peroxisomes, lipid metabolism, and peroxisomal disorders. Mol. Genet. Metab..

[35] Folz S.J., Trobe J.D. (1991). The peroxisome and the eye. Surv. Ophthalmol..

[36] Kang Q., Yang C. (2020). Oxidative stress and diabetic retinopathy: Molecular mechanisms, pathogenetic role and therapeutic implications. Redox Biol..

[37] Levin L.A., Gordon L.K. (2002). Retinal ganglion cell disorders: Types and treatments. Prog. Retin. Eye Res..

[38] Galindo-Camacho R.M., Blanco-Llamero C., da Ana R., Fuertes M.A., Senorans F.J., Silva A.M., Garcia M.L., Souto E.B. (2022). Therapeutic Approaches for Age-Related Macular Degeneration. Int. J. Mol. Sci..

[39] Ajith T.A. (2020). Alpha-lipoic acid: A possible pharmacological agent for treating dry eye disease and retinopathy in diabetes. Clin. Exp. Pharmacol. Physiol..

[40] Stirban A., Negrean M., Stratmann B., Gawlowski T., Horstmann T., Götting C., Kleesiek K., Mueller-Roesel M., Koschinsky T., Uribarri J. (2006). Benfotiamine Prevents Macro- and Microvascular Endothelial Dysfunction and Oxidative Stress Following a Meal Rich in Advanced Glycation End Products in Individuals with Type 2 Diabetes. Diabetes Care.

[41] Choudhary M., Ismail E.N., Yao P.L., Tayyari F., Radu R.A., Nusinowitz S., Boulton M.E., Apte R.S., Ruberti J.W., Handa J.T. (2020). LXRs regulate features of age-related macular degeneration and may be a potential therapeutic target. JCI Insight.

[42] Dhingra A., Sharp R.C., Kim T., Popov A.V., Ying G.-S., Pietrofesa R.A., Park K., Christofidou-Solomidou M., Boesze-Battaglia K. (2021). Assessment of a Small Molecule Synthetic Lignan in Enhancing Oxidative Balance and Decreasing Lipid Accumulation in Human Retinal Pigment Epithelia. Int. J. Mol. Sci..

[43] Zhang Q., Presswalla F., Ali R.R., Zacks D.N., Thompson D.A., Miller J.M.L. (2021). Pharmacologic activation of autophagy without direct mTOR inhibition as a therapeutic strategy for treating dry macular degeneration. Aging.

[44] Noguer M.T., Martinez M. (2010). Visual follow-up in peroxisomal-disorder patients treated with docosahexaenoic Acid ethyl ester. Investig. Ophthalmol. Vis. Sci..

[45] Toomey C.B., Kelly U., Saban D.R., Bowes Rickman C. (2015). Regulation of age-related macular degeneration-like pathology by complement factor H. Proc. Natl. Acad. Sci. USA.

[46] Cheung L.K., Eaton A. (2013). Age-related macular degeneration. Pharmacotherapy.

[47] Qamar Z., Qizilbash F.F., Iqubal M.K., Ali A., Narang J.K., Ali J., Baboota S. (2019). Nano-Based Drug Delivery System: Recent Strategies for the Treatment of Ocular Disease and Future Perspective. Recent Pat. Drug Deliv. Formul..

[48] Patel P., Shastri D., Shelat P., Shukla A. (2010). Ophthalmic drug delivery system: Challenges and approaches. Syst. Rev. Pharm..

[49] Tangri P., Khurana S. (2011). Basics of ocular drug delivery systems. Int. J. Res. Pharm. Biomed. Sci..

[50] Grassiri B., Zambito Y., Bernkop-Schnürch A. (2021). Strategies to prolong the residence time of drug delivery systems on ocular surface. Adv. Colloid Interface Sci..

[51] Fernandes A.R., Vidal L.B., Sánchez-López E., dos Santos T., Granja P.L., Silva A.M., Garcia M.L., Souto E.B. (2022). Customized cationic nanoemulsions loading triamcinolone acetonide for corneal neovascularization secondary to inflammatory processes. Int. J. Pharm..

[52] Sánchez-López E., Egea M.A., Cano A., Espina M., Calpena A.C., Ettcheto M., Camins A., Souto E.B., Silva A.M., García M.L. (2016). PEGylated PLGA nanospheres optimized by design of experiments for ocular administration of dexibuprofen—In vitro, ex vivo and in vivo characterization. Colloids Surf. B Biointerfaces.

[53] Sanchez-Lopez E., Egea M.A., Davis B.M., Guo L., Espina M., Silva A.M., Calpena A.C., Souto E.M.B., Ravindran N., Ettcheto M. (2018). Memantine-Loaded PEGylated Biodegradable Nanoparticles for the Treatment of Glaucoma. Small.

[54] Sanchez-Lopez E., Esteruelas G., Ortiz A., Espina M., Prat J., Munoz M., Cano A., Calpena A.C., Ettcheto M., Camins A. (2020). Dexibuprofen Biodegradable Nanoparticles: One Step Closer towards a Better Ocular Interaction Study. Nanomaterials.

[55] López-Machado A., Díaz-Garrido N., Cano A., Espina M., Badia J., Baldomà L., Calpena A.C., Souto E.B., García M.L., Sánchez-López E. (2021). Development of Lactoferrin-Loaded Liposomes for the Management of Dry Eye Disease and Ocular Inflammation. Pharmaceutics.

[56] Galindo R., Sánchez-López E., Gómara M.J., Espina M., Ettcheto M., Cano A., Haro I., Camins A., García M.L. (2022). Development of Peptide Targeted PLGA-PEGylated Nanoparticles Loading Licochalcone-A for Ocular Inflammation. Pharmaceutics.

[57] Souto E.B., de Souza A.L.R., Dos Santos F.K., Sanchez-Lopez E., Cano A., Zielińska A., Staszewski R., Karczewski J., Gremião M.P.D., Chorilli M. (2021). Lipid Nanocarriers for Hyperproliferative Skin Diseases. Cancers.

[58] De Oliveira I.F., Barbosa E.J., Peters M.C.C., Henostroza M.A.B., Yukuyama M.N., Dos Santos Neto E., Löbenberg R., Bou-Chacra N. (2020). Cutting-edge advances in therapy for the posterior segment of the eye: Solid lipid nanoparticles and nanostructured lipid carriers. Int. J. Pharm..

[59] Fangueiro J.F., Andreani T., Egea M.A., Garcia M.L., Souto S.B., Silva A.M., Souto E.B. (2014). Design of cationic lipid nanoparticles for ocular delivery: Development, characterization and cytotoxicity. Int. J. Pharm..

[60] Fangueiro J.F., Calpena A.C., Clares B., Andreani T., Egea M.A., Veiga F.J., Garcia M.L., Silva A.M., Souto E.B. (2016). Biopharmaceutical evaluation of epigallocatechin gallate-loaded cationic lipid nanoparticles (EGCG-LNs): In vivo, in vitro and ex vivo studies. Int. J. Pharm..

[61] Xu Y., Fourniols T., Labrak Y., Préat V., Beloqui A., des Rieux A. (2022). Surface Modification of Lipid-Based Nanoparticles. ACS Nano.

[62] Jacob S., Nair A.B., Shah J., Gupta S., Boddu S.H.S., Sreeharsha N., Joseph A., Shinu P., Morsy M.A. (2022). Lipid Nanoparticles as a Promising Drug Delivery Carrier for Topical Ocular Therapy—An Overview on Recent Advances. Pharmaceutics.

[63] Leonardi A., Bucolo C., Drago F., Salomone S., Pignatello R. (2015). Cationic solid lipid nanoparticles enhance ocular hypotensive effect of melatonin in rabbit. Int. J. Pharm..

[64] Ryals R.C., Patel S., Acosta C., McKinney M., Pennesi M.E., Sahay G. (2020). The effects of PEGylation on LNP based mRNA delivery to the eye. PLoS ONE.

[65] Eid H.M., Elkomy M.H., El Menshawe S.F., Salem H.F. (2019). Development, Optimization, and In Vitro/In Vivo Characterization of Enhanced Lipid Nanoparticles for Ocular Delivery of Ofloxacin: The Influence of Pegylation and Chitosan Coating. AAPS PharmSciTech.

[66] Singh D., Srivastava S., Pradhan M., Kanwar J.R., Singh M.R. (2015). Inflammatory bowel disease: Pathogenesis, causative factors, issues, drug treatment strategies, and delivery approaches. Crit. Rev. Ther. Drug Carr. Syst..

[67] Wang Y., Rajala A., Rajala R.V.S. (2015). Lipid Nanoparticles for Ocular Gene Delivery. J. Funct. Biomater..

[68] Moiseev R.V., Kaldybekov D.B., Filippov S.K., Radulescu A., Khutoryanskiy V.V. (2022). Maleimide-Decorated PEGylated Mucoadhesive Liposomes for Ocular Drug Delivery. Langmuir.

[69] Tavakoli S., Puranen J., Bahrpeyma S., Lautala V.E., Karumo S., Lajunen T., del Amo E.M., Ruponen M., Urtti A. (2022). Liposomal sunitinib for ocular drug delivery: A potential treatment for choroidal neovascularization. Int. J. Pharm..

[70] Chen X., Wu J., Lin X., Wu X., Yu X., Wang B., Xu W. (2022). Tacrolimus Loaded Cationic Liposomes for Dry Eye Treatment. Front. Pharmacol..

[71] Andra V.V.S.N.L., Pammi S.V.N., Bhatraju L.V.K.P., Ruddaraju L.K. (2022). A Comprehensive Review on Novel Liposomal Methodologies, Commercial Formulations, Clinical Trials and Patents. BioNanoScience.

[72] Jia M., Deng C., Luo J., Zhang P., Sun X., Zhang Z., Gong T. (2018). A novel dexamethasone-loaded liposome alleviates rheumatoid arthritis in rats. Int. J. Pharm..

[73] Hirsch M., Ziroli V., Helm M., Massing U. (2009). Preparation of small amounts of sterile siRNA-liposomes with high entrapping efficiency by dual asymmetric centrifugation (DAC). J. Control. Release.

[74] Souto E.B., Cano A., Martins-Gomes C., Coutinho T.E., Zielińska A., Silva A.M. (2022). Microemulsions and Nanoemulsions in Skin Drug Delivery. Bioengineering.

[75] Wang R., Gao Y., Liu A., Zhai G. (2021). A review of nanocarrier-mediated drug delivery systems for posterior segment eye disease: Challenges analysis and recent advances. J. Drug Target..

[76] Valizadehderakhshan M., Shahbazi A., Kazem-Rostami M., Todd M.S., Bhowmik A., Wang L. (2021). Extraction of cannabinoids from *Cannabis sativa* L. (Hemp). Agriculture.

[77] Herdiana Y., Wathoni N., Shamsuddin S., Muchtaridi M. (2022). Scale-up polymeric-based nanoparticles drug delivery systems: Development and challenges. OpenNano.

[78] Gonzalez-Pizarro R., Silva-Abreu M., Calpena A.C., Egea M.A., Espina M., García M.L. (2018). Development of fluorometholone-loaded PLGA nanoparticles for treatment of inflammatory disorders of anterior and posterior segments of the eye. Int. J. Pharm..

[79] Silva M., Peng T., Zhao X., Li S., Farhan M., Zheng W. (2021). Recent trends in drug-delivery systems for the treatment of diabetic retinopathy and associated fibrosis. Adv. Drug Deliv. Rev..

[80] Araujo J., Vega E., Lopes C., Egea M.A., Garcia M.L., Souto E.B. (2009). Effect of polymer viscosity on physicochemical properties and ocular tolerance of FB-loaded PLGA nanospheres. Colloids Surf. B Biointerfaces.

[81] Abrego G., Alvarado H., Souto E.B., Guevara B., Bellowa L.H., Parra A., Calpena A., Garcia M.L. (2015). Biopharmaceutical profile of pranoprofen-loaded PLGA nanoparticles containing hydrogels for ocular administration. Eur. J. Pharm. Biopharm..

[82] Li N., Zhao Z., Ma H., Liu Y., Nwafor E.-O., Zhu S., Jia L., Pang X., Han Z., Tian B. (2022). Optimization and Characterization of Low-Molecular-Weight Chitosan-Coated Baicalin mPEG-PLGA Nanoparticles for the Treatment of Cataract. Mol. Pharm..

[83] Wei S., Li J., He H., Shu C., Dardik A., Bai H. (2022). A three-layered hydrogel patch with hierarchy releasing of PLGA nanoparticle drugs decrease neointimal hyperplasia. Smart Mater. Med..

[84] Vega E., Egea M.A., Calpena A.C., Espina M., García M.L. (2012). Role of hydroxypropyl-β-cyclodextrin on freeze-dried and gamma-irradiated PLGA and PLGA–PEG diblock copolymer nanospheres for ophthalmic flurbiprofen delivery. Int. J. Nanomed..

[85] Vasconcelos A., Vega E., Pérez Y., Gómara M.J., García M.L., Haro I. (2015). Conjugation of cell-penetrating peptides with poly(lactic-co-glycolic acid)-polyethylene glycol nanoparticles improves ocular drug delivery. Int. J. Nanomed..

[86] Jiang K., Fan X., Hu Y., Yao S., Liu Y., Zhan C., Lu W., Wei G. (2022). Topical instillation of cell-penetrating peptide-conjugated melphalan blocks metastases of retinoblastoma. Biomaterials.

[87] Liu C., Tai L., Zhang W., Wei G., Pan W., Lu W. (2014). Penetratin, a potentially powerful absorption enhancer for noninvasive intraocular drug delivery. Mol. Pharm..

[88] Tai L., Liu C., Jiang K., Chen X., Feng L., Pan W., Wei G., Lu W. (2017). A novel penetratin-modified complex for noninvasive intraocular delivery of antisense oligonucleotides. Int. J. Pharm..

[89] Shipunova V.O., Sogomonyan A.S., Zelepukin I.V., Nikitin M.P., Deyev S.M. (2021). PLGA Nanoparticles Decorated with Anti-HER2 Affibody for Targeted Delivery and Photoinduced Cell Death. Molecules.

[90] Aktaş Y., Yemisci M., Andrieux K., Gürsoy R.N., Alonso M.J., Fernandez-Megia E., Novoa-Carballal R., Quiñoá E., Riguera R., Sargon M.F. (2005). Development and brain delivery of chitosan-PEG nanoparticles functionalized with the monoclonal antibody OX26. Bioconjugate Chem..

[91] Singh I.R., Pulikkal A.K. (2022). Preparation, stability and biological activity of essential oil-based nano emulsions: A comprehensive review. OpenNano.

[92] Ozogul Y., Karsli G.T., Durmuş M., Yazgan H., Oztop H.M., McClements D.J., Ozogul F. (2022). Recent developments in industrial applications of nanoemulsions. Adv. Colloid Interface Sci..

[93] Wilson R.J., Li Y., Yang G., Zhao C.-X. (2022). Nanoemulsions for drug delivery. Particuology.

[94] Kambhampati S.P., Kannan R.M. (2013). Dendrimer nanoparticles for ocular drug delivery. J. Ocul. Pharmacol. Ther..

[95] Marano R.J., Toth I., Wimmer N., Brankov M., Rakoczy P.E. (2005). Dendrimer delivery of an anti-VEGF oligonucleotide into the eye: A long-term study into inhibition of laser-induced CNV, distribution, uptake and toxicity. Gene Ther..

[96] Ghezzi M., Ferraboschi I., Delledonne A., Pescina S., Padula C., Santi P., Sissa C., Terenziani F., Nicoli S. (2022). Cyclosporine-loaded micelles for ocular delivery: Investigating the penetration mechanisms. J. Control. Release.

[97] Durgun M.E., Güngör S., Özsoy Y. (2020). Micelles: Promising Ocular Drug Carriers for Anterior and Posterior Segment Diseases. J. Ocul. Pharmacol. Ther..

[98] Tanito M., Hara K., Takai Y., Matsuoka Y., Nishimura N., Jansook P., Loftsson T., Stefánsson E., Ohira A. (2011). Topical dexamethasone-cyclodextrin microparticle eye drops for diabetic macular edema. Investig. Ophthalmol. Vis. Sci..

[99] Zaghloul N., Mahmoud A.A., Elkasabgy N.A., El Hoffy N.M. (2022). PLGA-modified Syloid(^®^)-based microparticles for the ocular delivery of terconazole: In-vitro and in-vivo investigations. Drug Deliv..

[100] Patel A., Cholkar K., Agrahari V., Mitra A.K. (2013). Ocular drug delivery systems: An overview. World J. Pharmacol..

[101] Tawfik S.M., Azizov S., Elmasry M.R., Sharipov M., Lee Y.-I. (2021). Recent Advances in Nanomicelles Delivery Systems. Nanomaterials.

[102] Vaishya R.D., Khurana V., Patel S., Mitra A.K. (2014). Controlled ocular drug delivery with nanomicelles. Wiley Interdiscip. Rev. Nanomed. Nanobiotechnol..

[103] Kumar D., Jain N., Gulati N., Nagaich U. (2013). Nanoparticles laden in situ gelling system for ocular drug targeting. J. Adv. Pharm. Technol. Res..

[104] Asma N., Maddeppungeng N.M., Raihan M., Erdiana A.P., Himawan A., Permana A.D. (2022). New HPLC-UV analytical method for quantification of metronidazole: Application to ex vivo ocular kinetic assessments following the administration of thermosensitive ocular in situ gel. Microchem. J..

[105] Bai L., Lei F., Luo R., Fei Q., Zheng Z., He N., Gui S. (2022). Development of a Thermosensitive In-Situ Gel Formulations of Vancomycin Hydrochloride: Design, Preparation, In Vitro and In Vivo Evaluation. J. Pharm. Sci..

